# Extensive downregulation of immune gene expression by microRNA-140-3p 5′ isomiR in an in vitro model of osteoarthritis

**DOI:** 10.1016/j.ocarto.2021.100189

**Published:** 2021-06-10

**Authors:** Rua Nader Al-Modawi, Jan E. Brinchmann, Tommy A. Karlsen

**Affiliations:** a Department of Immunology, Institute of Clinical Medicine, University of Oslo, Norway; b Department of Immunology and Transfusion Medicine, Oslo University Hospital, Norway; c Department of Molecular Medicine, Institute of Basic Medical Sciences, University of Oslo, Norway

**Keywords:** miR-140-3p, IsomiRs, Osteoarthritis, Immune regulation, HLA

## Abstract

**Objective:**

MicroRNA-140-3p is the most prevalent form of canonical miR-140 in native chondrocytes. IsomiRs are sequence variants of microRNAs with potentially distinct functionalities. Here we present functional studies of canonical microRNA-140-3p and two of its most prevalent isomiRs, a 5′ isomiR and a 3′ isomiR, in an inflammation-induced model of osteoarthritis (OA).

**Method:**

Canonical miR-140-3p, the 5′ isomiR and the 3′ isomiR were overexpressed separately in chondrocytes from three donors and subsequently subjected to an inflammatory milieu mediated by interleukin 1 beta and tumor necrosis factor alpha. RNA sequencing was performed on the cells to investigate the altered transcriptomes, RT-qPCR was performed to validate important observations, and western blot analysis was carried out to further study key inflammatory molecules.

**Results:**

The three microRNAs downregulated many of the same genes. However, the 5′ isomiR showed a much greater target spectrum compared to the other two miRNAs, and downregulated cascades of genes downstream of interferon beta, interferon gamma and interleukin 1 beta as well as genes involved in several other inflammatory and antiviral pathways. In addition the 5′ isomiR downregulated practically all HLA class II and class I genes.

**Conclusion:**

Introduction of the 5′ isomiR led to downregulation of genes essential for some of the most important inflammation cascades and virtual silencing of genes responsible for antigen presentation. These observations may indicate a very promising therapeutic potential for the 5′ isomiR for OA and several inflammatory conditions, particularly HLA associated immune conditions including many arthritic diseases.

## 1 Introduction

Inflammation, mediated in part through the key inflammatory cytokines interleukin 1 beta (IL1β) and tumor necrosis factor alpha (TNFα), is an important driver in the pathogenesis of OA [1,2], as it promotes the degradation of existing extracellular matrix (ECM) [[2], [3], [4]] and inhibits synthesis of new ECM [5].

Recent research points to the involvement of microRNAs (miRNAs) in these processes [[6], [7], [8], [9], [10]]. miRNAs are small non-coding RNAs that regulate gene expression. Following transcription, processing and export from the nucleus they are found in the cytoplasm as mature miRNA duplexes 20–25 nucleotides (nt) long. One or both of the strands are then incorporated into the RNA-induced silencing complex (RISC) [11]. Within the RISC the miRNA binds to its target mRNA through base-pairing between the so-called seed sequence (nucleotide 2–8) of the miRNA and the 3′ UTR of the mRNA [12,13]. This leads to degradation and/or translational repression of the mRNA [14,15].

miR-140 has been considered a cartilage specific miRNA since it was observed to be predominantly expressed in cartilaginous tissue during development [16]. Knockout studies showed miR-140 to be protective against OA development [6]. Both miR-140-5p and miR-140-3p are highly upregulated during in vitro chondrogenesis [8], and we previously showed that both strands are highly expressed in healthy cartilage, miR-140-3p higher than miR-140-5p [17]. We also showed that miR-140-5p was essential for SOX9 expression during in vitro chondrogenesis [8], and demonstrated anti-inflammatory protective effects of both miR-140-5p and miR-140-3p on chondrocytes in two different in vitro models simulating OA [9,10].

The repertoire of miRNAs is increasing in complexity as recent deep sequencing studies have revealed the existence of many sequence variations in addition to the canonical sequences [18,19]. These variants are called isomiRs. The sequence variation can be an addition or a deletion of one or more nucelotides at the 5′ and/or 3′ ends giving rise to 5′ or 3′ isomiRs. A substitution of a nucleotide gives rise to polymorphic isomiRs. IsomiRs are generated by RNA editing, alternative Drosha or Dicer processing, exonuclease mediated nucleotide trimming and/or non templated nucleotide addition [18,20]. 5′ isomiRs will have a different seed sequence from the canonical miRNA, and this may alter target recognition considerably [21,22]. Recently the miRNA and isomiR prevalence in briefly cultured articular chondrocytes was published by Haseeb and colleagues [23]. miR-140-3p was found to have the highest number of isomiRs, and several of these were found at higher prevalence than the canonical miR-140-3p. Another recent report also showed that miR-140-3p isomiRs were functional and regulated many other genes than canonical miR-140-3p [24]. Here we show how the canonical miR-140-3p and two of its most prevalent isomiRs, one 5′ and one 3′ isomiR, vary in their effect on mRNA expression in articular chondrocytes in an inflammation-induced model of OA. The results showed that the three miRNAs overlapped in their regulation of the same biological processes, all with a predominantly anti-inflammatory effect. The 5′ isomiR, which by far downregulated the greatest number of mRNAs, showed extensive downregulation of genes involved in a number of immune response pathways.

## 2 Methods and materials

### 2.1 Isolation and culture of human articular chondrocytes (ACs)

ACs were isolated from discarded OA cartilage tissue after total knee replacement surgery and cultured as previously described [17]. Only tissue with no macroscopic signs of OA was used. All donors provided written, informed consent. The study was approved by the Regional Committee for Medical Research Ethics, Southern Norway. Briefly, the cartilage was cut into miniscule pieces and subsequently digested with Collagenase type XI (Sigma-Aldrich, St. Louis, MO) at 37 ​°C for 90–120 ​min. Chondrocytes were washed three times and resuspended in culture medium consisting of Dulbecco's modified Eagle's medium/F12 (Gibco/ThermoFisher Scientific, Waltham, MA, USA) supplemented with 10% human plasma (Octapharma AB, Oslo Blood Bank, Norway) supplemented with platelet lysate (corresponding to 10^9^ platelets/ml plasma) (PLP), 100 units/mL penicillin, 100 ​μg/mL streptomycin, and 2.5 ​μg/mL amphotericin B [17]. PLP was prepared as previously described [25]. The culture medium was changed every 3–4 days. After the first passage amphotericin B was removed. At 70–80% confluence, cells were detached with trypsin-EDTA (Sigma-Aldrich) and seeded into new culture flasks.

### 2.2 MicroRNA mimics, transfection and stimulation with IL1β and TNFα

The Amaxa nucleofector system and the Amaxa Human Chondrocyte Nucleofector Kit were used for electroporation following the protocols from the manufacturer (Lonza, Walkersville, MD). Briefly, each reaction contained 1.0 ​× ​10^6^ ​cells, 5 ​μM of miRvana mimics (Supplementary Table S8) in a total volume of 100 ​μl nucleofection solution. The cells were seeded in 20% PLP without antibiotics and left to recover over night. The following day (day 1) the medium was changed to 10% PLP with 1% penicillin/streptomycin. On day 4 ​ACs were stimulated with 0.1 ​ng/mL recombinant IL1β (IL1β) and 10 ​ng/mL TNFα (R&D Systems, Minneapolis, MN) for 24 ​h before harvesting for analysis.

### 2.3 Isolation of miRNA, cDNA synthesis, and RT-qPCR

Total RNA containing miRNAs was isolated using the miRNeasy mini kit according to manufacturer's protocol (Qiagen, Germantown, MD). cDNA synthesis and RT-qPCR were performed following protocols from the manufacturer using the Taqman High capacity cDNA Reverse Transcription Kit for mRNA and Taqman MicroRNA Reverse Transcription Kit for microRNAs and the Taqman™ Universal PCR Master Mix (Thermo Fisher Scientific, Waltham, MA, USA). Relative quantification (ΔΔCT) was used for quantification of mRNA levels between samples. 2 ​ng miRNA in a total volume of 15 ​μl, and for other genes 200 ​ng RNA in a total volume of 15 ​μl was reverse transcribed into cDNA. All samples were run in technical triplicates. Each replicate contained 1.33 ​μl cDNA in a total volume of 15 ​μl for miRNAs and 0.2 ​μl cDNA in a total volume of 15 ​μl for mRNAs. The thermocycling parameters were 95 ​°C for 10 ​min followed by 40 cycles of 95 ​°C for 15 ​s and 60 ​°C for 1 ​min. U6 was used as endogenous control for miRNAs and GAPDH was used as endogenous control for mRNAs. RT-qPCR results are shown as relative fold changes using mean values from technical triplicates with a 95% confidence interval. All donors are shown separately in the figures.

### 2.4 Western blotting

Cell lysates corresponding to 200,000 ​cells were loaded onto a 4–20% gradient or 10% polyacrylamide gel (Bio-Rad, Hercules, CA). Proteins were separated by gel electrophoresis, transferred to PVDF membranes and incubated with appropriate antibodies (Suppelemntary Table S10) before visualizing the bands using the myECL imager (Thermo Fisher Scientific).

### 2.5 RNA-sequencing

Sequence libraries from mRNA was prepared using the TruSeq Stranded mRNA kit (Illumina) at the Norwegian Sequencing Center, Oslo University Hospital, Ullevål. BBMap v34.56 was used to remove low quality reads and adapter sequences. HiSat2 v2.1.0 for mapping reads to the genome and Samtools v1.2 to convert SAM files to BAM files. The BAM files was uploaded and analyzed using the Seqmonk software (https://www.bioinformatics.babraham.ac.uk/). The DEseq2 package in Seqmonk was used for differential expression analysis. To correct for library size and RNA composition bias DEseq2 uses an internal normalization where the geometric mean is calculated for each gene across all the samples. For each sample the counts for a gene is divided by the geometric mean and the median of these ratios in a sample is the size factor for that sample. ShinyGO v0.61 was used for GO-term and Gene set enrichment analysis and for in silico prediction of transcription factor-binding sites [26].

### 2.6 3UTR Luciferase assay

LentiX cells were co-transfected with plasmids and a negative control or 5′ isomiR mimic using Lipofectamine 2000. The next day Luciferase activity were measured on BioTek Synergy H1 multimode platereader (Thermo Fisher Scientific).

### 2.7 Statistics

One-way ANOVA with Bonferroni correction was used for all RT-qPCR analysis and luciferase analysis using the Graphpad Prism Software. ∗ ​= ​P value less than 0.05 after multiple correction.

## 3 Results

**Fig. 1:**
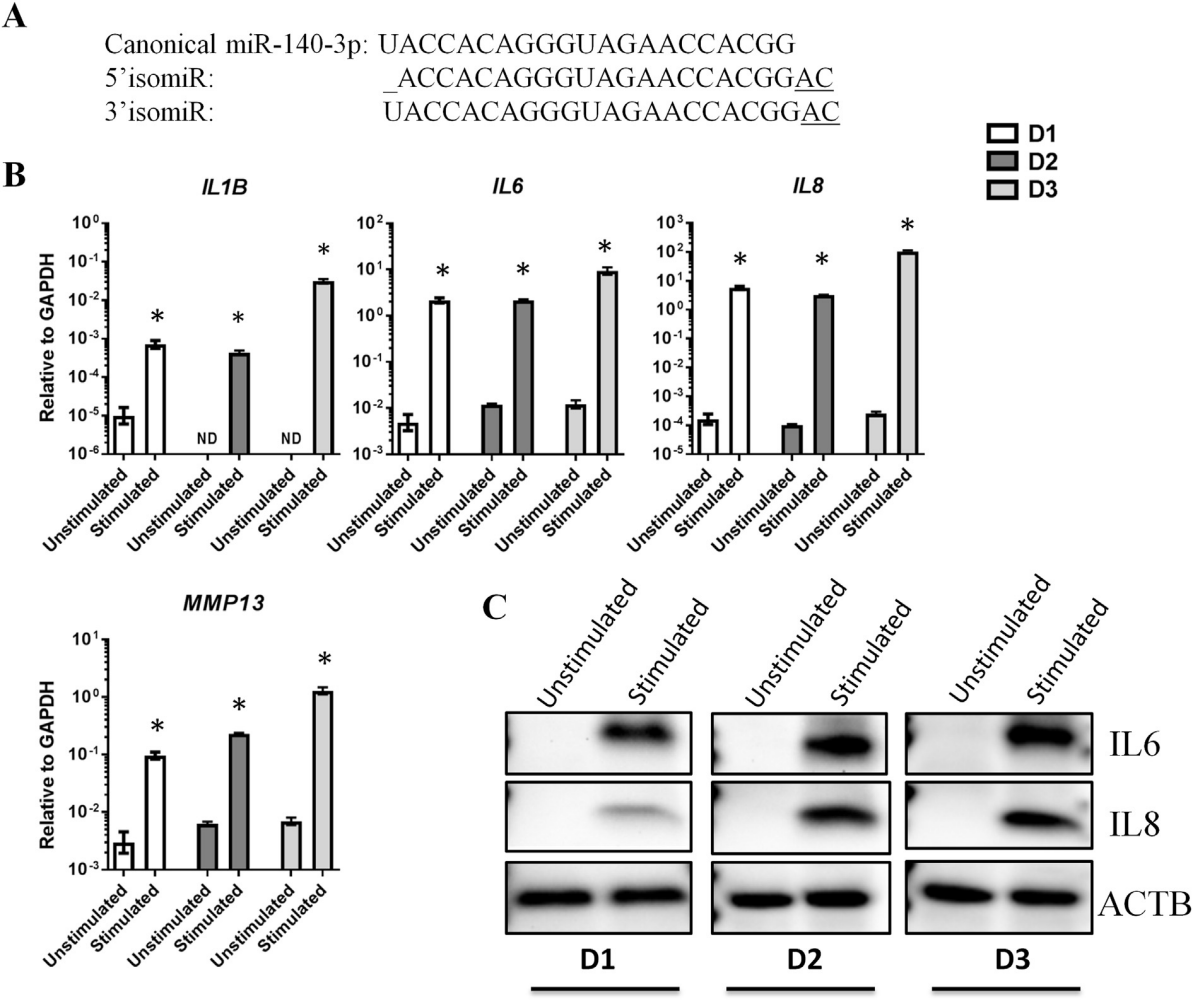
**Upregulation of OA mediators in response to IL1β and TNFα**. **(A)** The sequences of the three miRNAs. (**B**) RT-qPCR analysis of *IL1B*, *IL6*, *IL8*, and *MMP1*3 mRNA levels in unstimulated and IL1β and TNFα-stimulated conditions in chondrocytes from 3 OA donors. Error bars represent a 95% confidence interval from technical triplicates. (**C**) Western blot analysis of IL6 and IL8 protein levels in unstimulated and IL1β and TNFα-stimulated conditions in the same donors. All samples derive from the same experiment and the blots were processed in parallel. β-actin (ACTB) was used as loading control. D1 ​= ​donor 1, D2 ​= ​donor 2, D3 ​= ​donor 3. ND ​= ​not detected. ∗ ​= ​P value less than 0.05 after multiple correction using One-way ANOVA with Bonferroni correction.

The sequences of the canonical miR-140-3p and our chosen 5′ and 3′ isomiR are shown in Fig. 1A. The 5′ isomiR was chosen because it was the sequence with the highest expression in articular chondrocytes in the study performed by Haseeb et al. [23], and it was also prevalent in our recent study of miRNAs and isomiRs in plasma extracellular vesicles both in patients with OA and controls [27,28]. It lacks the first nt of the canonical miR-140-3p, and has two additional nt at the 3′ end, A and C. The 3′ isomiR was chosen because it has the same two additional nt at the 3′ end, and is otherwise identical to the canonical sequence. The 3′ isomiR, too, is prevalent in the Haseeb et al. study and in our own isomiR analyses.

An inflammation-induced model using cytokines found in OA was established in vitro by stimulating cultured articular chondrocytes with IL1β and TNFα. IL1β and TNFα strongly upregulated the mRNA expression of the OA-inducing cytokines *IL1B*, *IL6*, *IL8* and the matrix degrading enzyme *MMP13* in cells from all donors (Fig. 1B). Fig. 1C shows induced protein levels of OA associated cytokines IL6 and IL8 in response to stimulation by IL1β and TNFα.

**Fig. 2:**
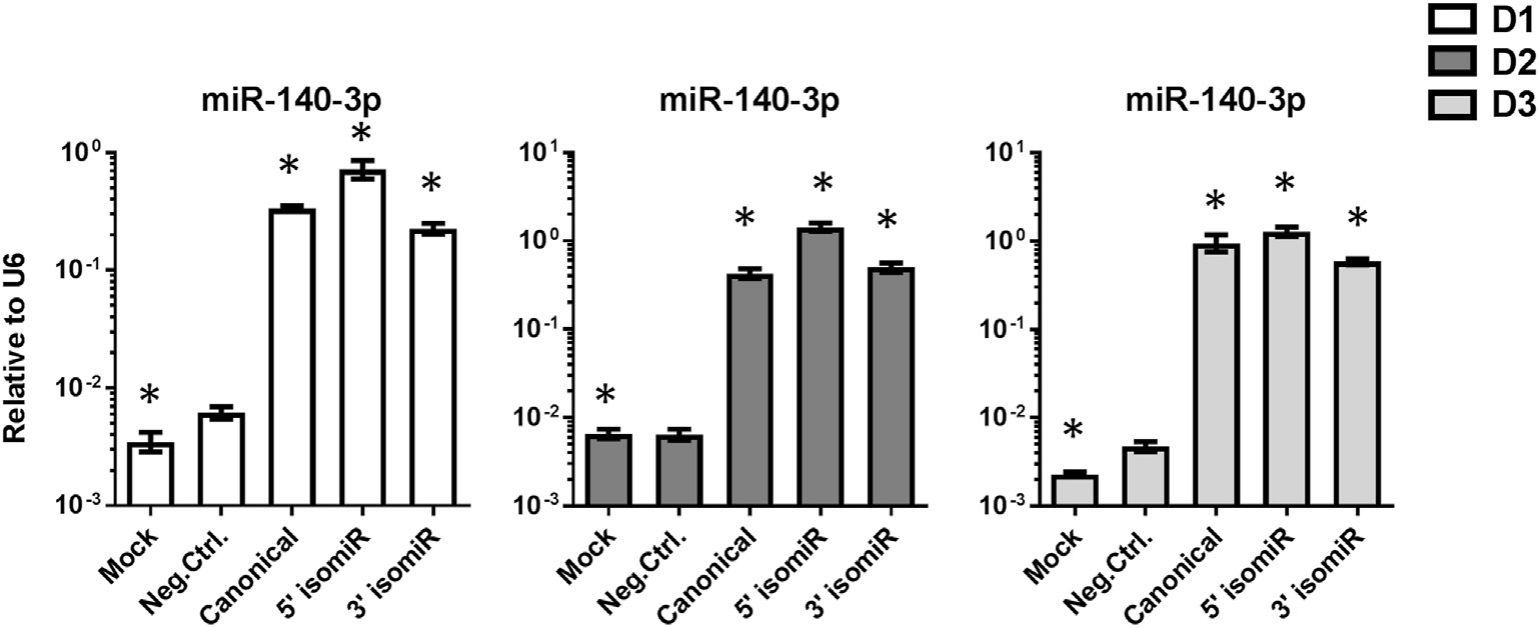
**Increased levels of miRNAs following transfection**. RT-qPCR analysis of canonical miR-140-3p, 5′ isomiR, and 3′ isomiR levels in chondrocytes from 3 donors after transfection. Error bars represent a 95% confidence interval from technical triplicates. IsomiRs were quantified with canonical miR-140-3p Taqman assay. D1 ​= ​donor 1, D2 ​= ​donor 2, D3 ​= ​donor 3. ∗ ​= ​P value less than 0.05 after multiple correction using One-way ANOVA with Bonferroni correction.

**Supplementary Fig. S1:**
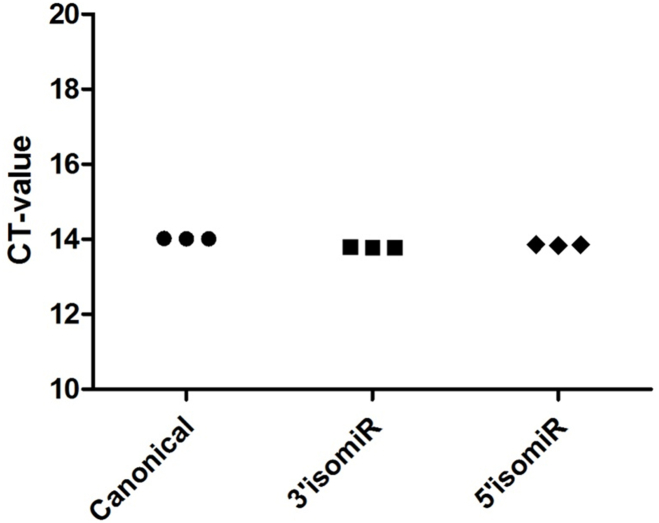
**Probe-testing.** RT-qPCR analysis of amplified miRNA mimics; canonical miR-140-3p, 3′ isomiR and 5′ isomiR using probe designed for the canonical sequence. RT-qPCR reaction was carried out on the synthetic mimics taken directly from tubes.

Our previous study showed that canonical miR-140-3p inhibited IL1β and TNFα-mediated inflammation [10]. To investigate the role of the 5′ isomiR and 3′ isomiR in OA compared to their canonical sequence, we transfected chondrocytes with each of the miRNAs separately. Higher levels of the miRNA sequences were confirmed in all three donors (Fig. 2). Mock (electroporated cells) and transfection of a negative control sequence were both used as controls. The Taqman MicroRNA assay used for RT-qPCR is designed for the canonical miR-140-3p sequence, but it amplified both isomiRs as well. This was tested for all three sequences by RT-qPCR amplification directly from the reagent tube (Supplementary Fig. S1).

### 3.1 miR-140-3p and its isomiRs have different effects on key inflammatory cytokines

**Fig. 3:**
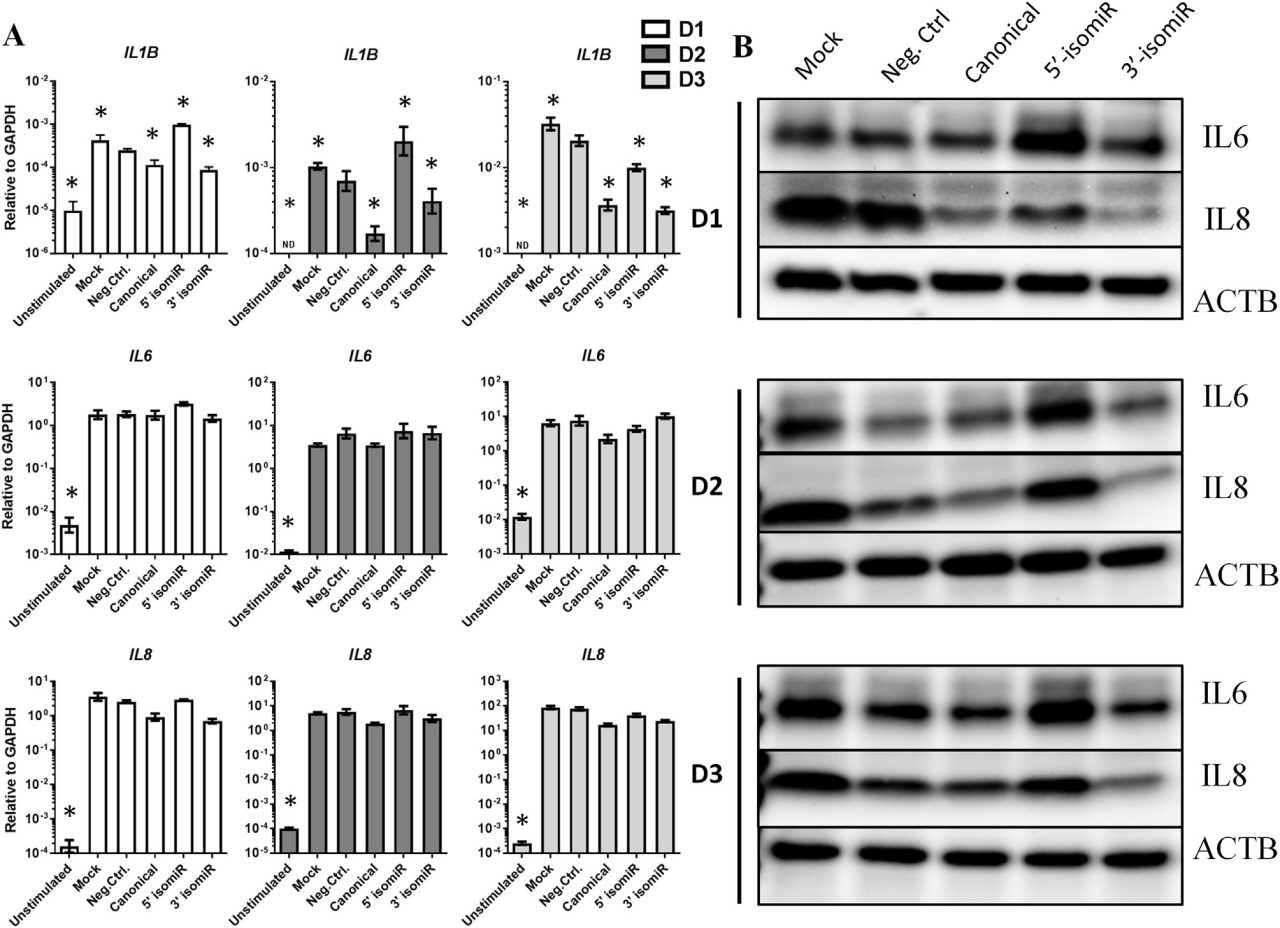
**Canonical miR-140-3p and 3′ isomiR counteracted IL1β and TNFα-induced inflammation, while 5′ isomiR showed opposing tendencies**. (**A**) RT-qPCR analysis of *IL1B, IL6*, and *IL8* mRNA levels after transfection of the three miRNAs. Error bars represent a 95% confidence interval from technical triplicates. (**B**) Western blot analysis of IL6 and IL8 in the same three donors. All samples derive from the same experiment and the blots were processed in parallel. ACTB was used as loading control. D1 ​= ​donor 1, D2 ​= ​donor 2, D3 ​= ​donor 3. ND ​= ​not detected. ∗ ​= ​P value less than 0.05 after multiple correction using One-way ANOVA with Bonferroni correction.

We then wanted to study the effect of the different miRNAs on key inflammatory cytokines within the inflammatory milieu mediated by IL1β and TNFα. Canonical miR-140-3p and its 3′ isomiR downregulated *IL1B* and, marginally, *IL8* (Fig. 3A). The 5′ isomiR upregulated IL6 and IL8 protein compared with canonical miR-140-3p and the 3′ isomiR, and practically always also compared with the negative control. At the protein level (Fig. 3B), one consistent observation was that IL6 and IL8 were both downregulated by the negative control relative to mock.

### 3.2 RNA sequencing revealed both unique and overlapping changes in gene expression following transfection of the canonical miR-140-3p or its isomiRs

**Fig. 4:**
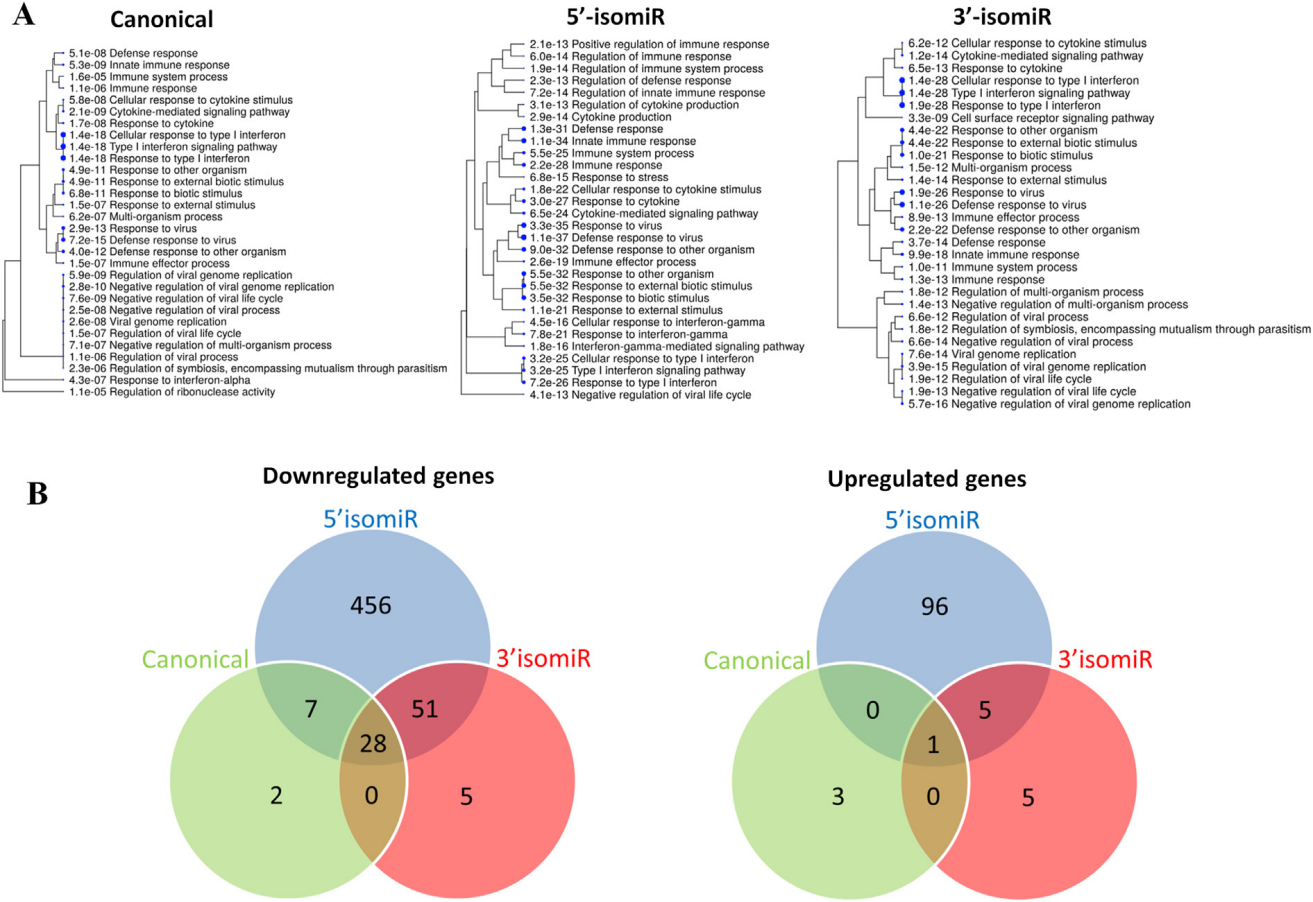
**RNA-sequencing data revealed many immunological processes to be targeted by all three miRNAs**. (**A**) Go-term analysis of the downregulated genes by canonical miR-140-3p, 5′ isomiR, and 3′ isomiR. The numbers in the figure are the FDR values for each term and the size of the blue dots correlates with significance **(B)** Venn diagram illustrating the number of common regulated genes by the miRNAs; downregulated and upregulated genes are shown separately.

In order to further unravel the biological impact of miR-140-3p and the two isomiRs we performed RNA sequencing on the same cells as were used to produce the results described in Fig. 3. The negative control sequence was used as control, as there were no significantly differentially expressed genes compared with mock control. Canonical miR-140-3p, 5′ isomiR and 3′ isomiR downregulated the expression of 37, 542, and 84 genes and upregulated the expression of 4, 102, and 11 genes, respectively (FDR <0.05, cut-off at 2 fold difference, Supplementary Tables S1–S3). Gene Ontology (GO)-analysis of downregulated genes showed that all three miRNAs regulated the same or related biological processes (Fig. 4A). Almost all GO-terms were related to immune responses such as type I interferon reponses, innate immunity response, defense responses virus and to other pathogens, suggesting that many of the same genes or gene families were targeted by the three miRNAs. And this was, indeed, the case. Having the same seed sequence as the canonical miR-140-3p, the 3′ isomir would be expected to have a similar spectrum of target mRNAs. We found that 28 of the 37 mRNAs (76%) downregulated by the canonical miR-140-3p were also downregulated by the 3′ isomiR (Fig. 4B, Supplementary Table S4). The 3′ isomiR also downregulated another 56 mRNAs, of which 51 (91%) were also downregulated by the 5′ isomiR. The 28 mRNAs that were downregulated by both the canonical and the 3′ isomiR were also downregulated by the 5′ isomiR. This left very few mRNAs, two and five, to be uniquely downregulated by the canonical miR-140-3p and its 3′ isomiR, respectively (Fig. 4B). Thus, most of the following results will be related to the downregulatory effect of the 5′ isomiR.

### 3.3 The 5′ isomiR downregulated cascades of immunologically active mRNAs

**Supplementary Fig. S2:**
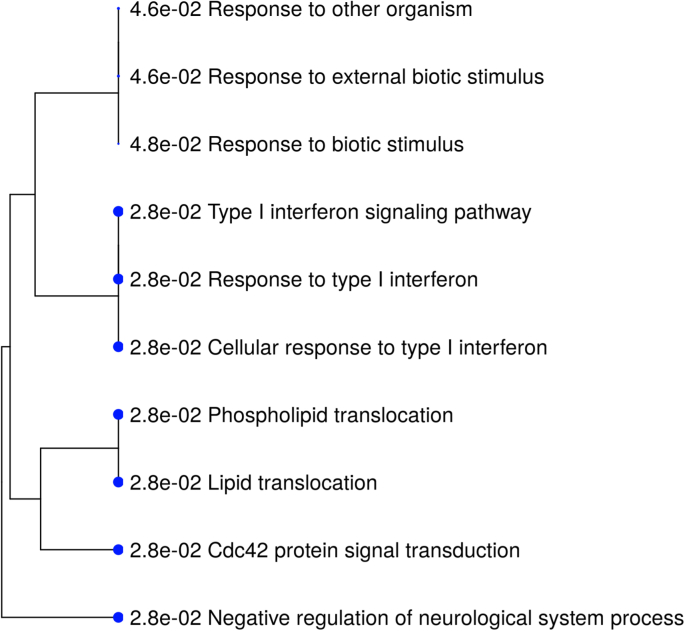
**GO-term.** GO-term analysis on the 27 genes that were both downregulated and predicted to be targets of 5′ isomiR.

**Supplementary Fig. S3:**
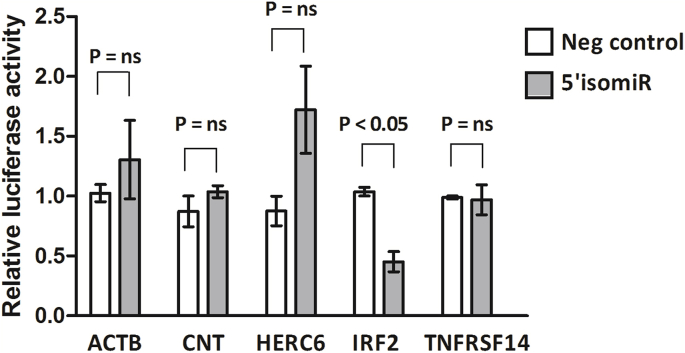
**Luciferase assay.** Luciferase activity after co-transfection of plasmids containing the 3UTR of indicated genes and with negative control or 5′ isomiR mimic. ∗ ​= ​P value less than 0.05 after multiple correction using One-way ANOVA with Bonferroni correction.

The miRDB database predicted 100 genes to be targeted by both canonical miR-140-3p and 5′ isomiR (Supplementary Table S5) suggesting several common targets despite having different seeds. For canonical and 3′ isomiR (same seed) 697 genes were predicted to be targets by the database. Only 3 genes that were downregulated by canonical (CMPK2, RASGEF1B and RNF213) and 2 genes by 3′ isomiR (CMPK2 and RNF213) were predicted targets. Among the 542 genes downregulated by the 5′ isomiR, the database predicted 27 to be targets of the 5′ isomiR (Supplementary Table S6). GO-term analysis of these genes showed that they were mainly involved in immune responses and lipid translocation (Supplementary Fig. S2) Each of these could, potentially, initiate cascades of downstream events leading to further downregulation of mRNAs. Scrutiny of the 27 predicted targets downregulated by the 5′ isomiR revealed three possible cascade initiating candidates: *IRF2*, *RNASE4* and *TNFRSF14*, (Supplementary Table S3.A). IRF2 was shown to be a direct target of 5′ isomiR using a luciferase assay (Supplementary Fig. S3).

**Fig. 5:**
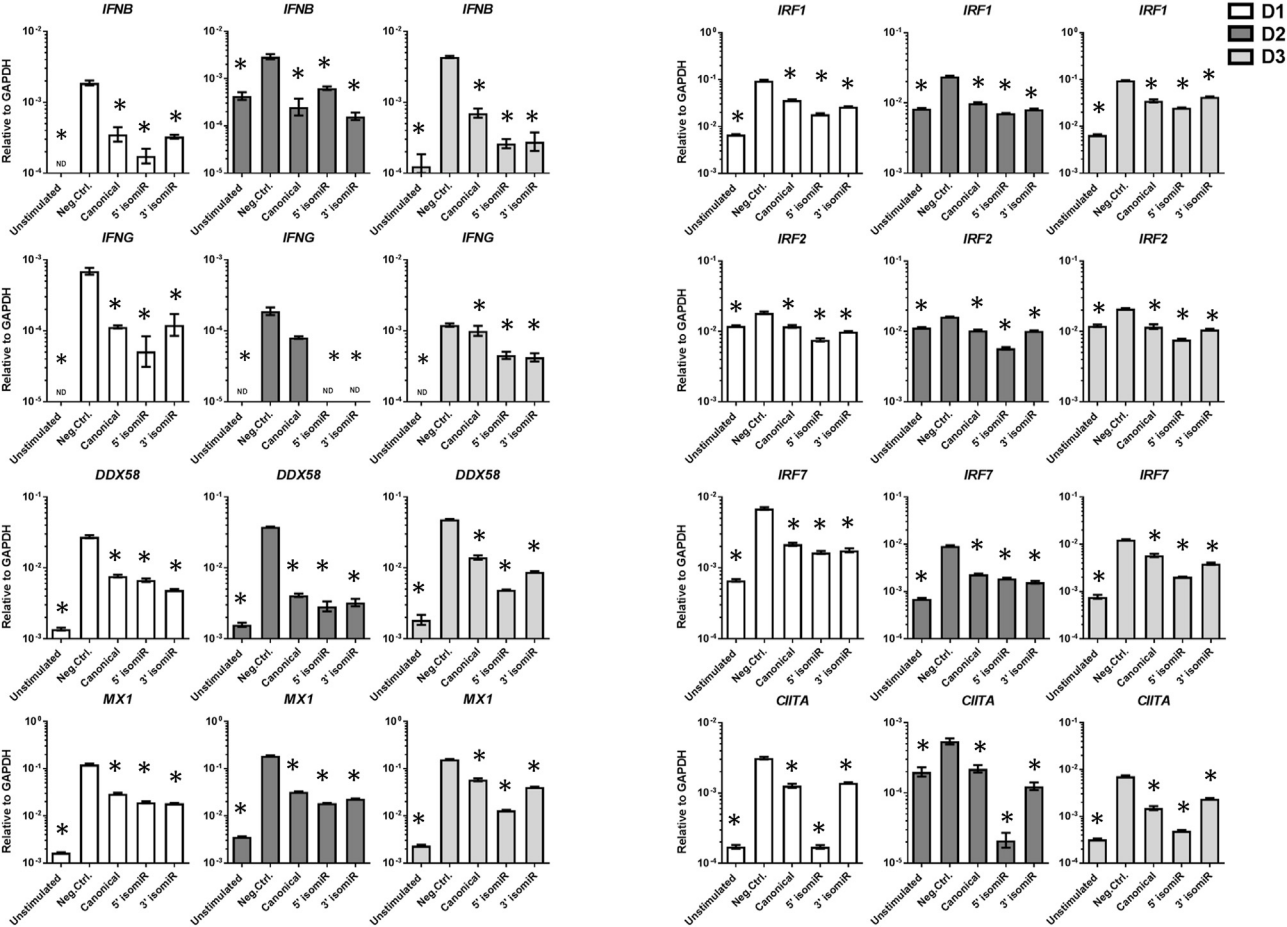
**RT-qPCR validation of RNA-seq data**. RT-qPCR analysis of *IFNB, IFNG*, *DDX58 MX1, IRF1,2* and *7, CIITA,* and after transfection of the three miRNAs. Error bars represent a 95% confidence interval from technical triplicates. Error bars represent a 95% confidence interval from technical triplicates. GAPDH was used as endogenous control. D1 ​= ​donor 1, D2 ​= ​donor 2, D3 ​= ​donor 3. ND ​= ​not detected. ∗ ​= ​P value less than 0.05 after multiple correction using One-way ANOVA with Bonferroni correction.

Examining the list of GO terms for evidence of cascades of downregulated mRNAs following 5′ isomiR transfection, the type I interferon (IFNA/IFNB) and interferon gamma (IFNG) pathways turned up as highly significant. Neither *IFNA* nor *IFNB* were detected to be significantly downregulated in the RNA seq analysis. However, RT-qPCR analysis showed that *IFNB* was in fact downregulated (Fig. 5), while *IFNA* was not detected. *IFNG* was downregulated by 32-fold (Supplementary Table 3.A, validated by PCR in Fig. 5). *IL12A* and *IL12RB1* are involved in the induction of *IFNG* transcription and were downregulated (Supplementary Table S3.A). *CASP1* is required for the conversion of pro-ILβ1 to IL1β [29] and was also downregulated (Supplementary Table S3.A). Another downregulated mRNA that is involved in IL1β signaling was MYD88 (Supplementary Table S3.A). Together this may explain the downregulation of molecules of the IL1β cascade.

Another cascade initiator downregulated by the 5′ isomiR is the cytoplasmic sensor of viral nucleic acids *DDX58* (also known as *RIG-1*), which activates a cascade of antiviral responses (Supplementary Table S3.A, validated in Fig. 5). *MX1* is another molecule that is involved in cellular anti-viral response and was also downregulated (Supplementary Table S3.A, validated in Fig. 5). Yet others are the transcription factors *IRF1*, *IRF2* and *IRF7* (Supplementary Table S3.A, validated in Fig. 5), and several cytokines.

Scrutiny of Supplementary Table S3.A shows another interesting series of downregulated genes: practically all genes and pseudogenes in the HLA class II histocompatibility antigens region and, to a lesser extent also the HLA class I region on chromosome 6 were downregulated: *HLA-DMA*, *HLA-DOB*, *HLA-DPA1*, *HLA-DPB1*, *HLA-DQA1*, *HLA-DQB1*, *HLA-DRB1*, *HLA-DRB5* as well as HLA class I genes *HLA-A*, *HLA-B*, *HLA-F*, *HLA-K,* but not the HLA class III region genes. In addition *TAP1* mRNA, encoding a protein which is essential for the availability of cytosolic peptides to HLA class I molecules in the endoplasmic reticulum, was also downregulated. The master regulator of the HLA class II region, Class II transactivator CIITA, is induced by IFNG [30]. To our surprise, *CIITA* was not downregulated by the 5′ isomiR according to our sequencing analysis. However, by RT-qPCR *CIITA* was found to be greatly downregulated to, or below, the level found in unstimulated chondrocytes (Fig. 5). Downregulation of *CIITA*, then, most likely explains the downregulation of the HLA class II mRNAs. Downregulated genes *IRF1* [31] and *AIM2* [32] may also regulate HLA class II expression as both act through CIITA.

For the HLA class I region the explanation probably lies in the downregulation of the master regulator, *NLRC5* mRNA (Supplementary Table S3.A) [33]. Although the majority of the genes downregulated by 5′ isomiR are positive regulators of inflammation and immune responses, two genes have been shown to be negative regulators of IL6, *SOCS3* [34,35] and *ZC3H12D* [36]. The downregulation of these two genes could perhaps explain why 5′ isomiR leads to upregulation of IL6 and IL8.

Interestingly, the promotors of the downregulated genes were enriched for DNA binding sites for several immunregulatory transcription factors, including *IRF7, IRF1* and *IRF2* that were also downregulated by all three miRNAs (Supplementary Table S7, Fig. 5). Thus downregulation of these transcription factors most likely contributes to downregulation of many of the genes in Supplementary Table S3.A.

In addition the cartilage and matrix associated genes *ACAN*, *HAPLN3*, *PRG4*, *FMOD*, *PRELP*, *CCN6/WISP3, ROR2, CEMIP*, *ADAMTS4*, *MMP1* and *MMP12* were also downregulated by 5′ isomiR. 5′ isomiR also targeted many non-coding genes that are either pseudogenes, non-coding or long non-coding RNA. In total 18.5% of the downregulated genes were non-protein coding genes.

### 3.4 Upregulated mRNAs

Only four genes were upregulated by canonical miR-140-3p and 11 genes were upregulated by the 3′ isomiR (Supplementary Table S1.B and S2.B respectively). 5′ isomiR transfection led to upregulation of 102 mRNAs, some of which are integrins, growth factors and matrix related enzymes (Supplementary Table S3.B). One gene, FZD6, was upregulated by all three miRNAs, while 45% (5 of 11) genes upregulated by 3′ isomiR were also upregulated by 5′ isomiR (Fig. 4B).

## 4 Discussion

miRNAs are potent regulatory molecules with interesting therapeutical potential for OA and other diseases. Their repertoire is only increasing in complexity with the emergence of deep sequencing data revealing numerous isomiRs of canonical miRNAs. In order to unleash that therapeutical potential more knowledge is required to understand how isomiRs operate together with or in comparison to their canonical sequences. This study aimed to unravel the role of the most prevalent cartilage miRNAs, miR-140-3p, and two of its isomiRs, in an inflammation-induced model using cytokines found in OA.

The 5′ isomiR downregulated by far the most mRNAs and a great majority of the downregulated genes were components of functionally interacting cascades, where the downregulation of one gene by the 5′ isomiR most likely led to the downregulation of a number of mRNAs in the same pathway. One such cascade is induced by IFNγ. *IFNG* is not constitutively expressed in chondrocytes as far as we know, and our RT-qPCR data support this claim. Also IL1β and TNFα are not known to induce the expression of IFNG, but these cytokines are shown here to do so. The synthesis of IFNγ as a consequence of IL1β and TNFα exposure will induce the expression of a cascade of molecules. In cells transfected with the 5′ isomiR *IFNG* was considerably downregulated, and RT-qPCR showed that the 3′ isomiR and canonical miR-140-3p also downregulated IFNG, albeit to a lesser extent. *IFNG* is not known to be a direct target of the 5′ isomiR. However, IL12A and IL12RB1 are involved in the induction of *IFNG* transcription, and their mRNAs are both downregulated suggesting that they, in part, may be responsible for the downregulation of IFNG mRNA following transfection of the 5′ isomiR. The IFNγ and IFNα/β signaling pathways are known to cross-talk at multiple levels [37], suggesting that downregulation of *IFNG* may reduce the level of mRNAs that are also classified to the IFNα/β pathway. Moreover RT-qPCR analysis showed that *IFNB* was also downregulated, which could affect several genes in the IFNα/β pathway.

Other cascade events are probably initiated by the downregulation of functional IL1β, other cytokines, *IRF*s 1, 2 and 7 and *DDX58*. Together these cascades make up inflammasomes generated by many different stimuli, and their downregulation may turn out to have very interesting therapeutic potential. Downregulation of these cascades probably account for the vast majority of downregulated mRNAs in this model system.

Another immunologically important effect of the overexpression of the 5′ isomiR is the downregulation of practically the entire HLA class II, as well as the HLA class I region on the short arm of chromosome 6 and *TAP1.* HLA class II and class I regions are transcriptionally regulated by master regulators and members of the NOD-like receptor family CIITA [38] and NLRC5 [33] (CITA), respectively. Both of these are induced by IFNγ [38,39], and both were shown to be downregulated by the 5′ isomiR, perhaps as a consequence of the downregulation if *IFNG*. The downregulated genes *IRF1* and *AIM2* may also regulate HLA class II expression. Both genes are also induced by IFNγ, and act through *CIITA* [31,32]. A molecule that inhibits the presentation of HLA class II and class I antigens may well turn out to have therapeutic potential, for instance in cases of HLA restricted autoimmune disease including many arthritic diseases.

The validation experiments with RT-qPCR mostly revealed close similarity between data obtained by RNA-seq and RT-qPCR. However, stringent statistical analysis might disqualify a gene from significance; for instance *DDX58* was significantly downregulated by 5′ isomiR and 3′ isomiR, but not by canonical miR-140-3p in the RNA-seq data, while RT-qPCR validated its downregulation by the canonical sequence. *CIITA* and *IFNB* were not detected by RNA-seq, however RT-qPCR showed their downregulation by all three miRNAs. These validation experiments also suggest that there are probably more genes regulated in common by all three miRNAs than what the RNA-seq data showed.

The 5′ isomiR also downregulated several cartilage and matrix related genes such as *ACAN*, the major component of cartilage, and *PRG4* (lubricin), together with *MMP1*, *MMP12* and *ADAMTS4* suggesting a role for 5′ isomiR also in extracellular matrix metabolism. However, in vivo the 5′ isomiR is expressed alongside other miR-140 sequences, notably miR-140-5p which has been shown to have a profound anabolic effect on cartilage matrix molecules. Thus, one may speculate that the miR-140-3p 5′ isomiR is important predominantly through its anti-inflammatory effect, while the downregulation of *ACAN* and *PRG4* are overruled by the upregulation of these molecules by the miR-140-5p [9].

Heterogenity at the 5′ end as a result of the inconsistent processing by Dicer and Drosha generate 5′ isomiRs with different seed sequences, which affects target recognition and regulation [21,22]. In the case of canonical miR-140-3p and the 5′ isomiR investigated in this study, one nt change at 5′ end led to the downregulation of 505 more genes compared to canonical miR-140-3p. Interestingly, 94% of the genes downregulated by canonical miR-140-3p were also downregulated by 5′ isomiR. Perhaps the two different seed sequences bind to different target sequences on the 3′ UTR of the same mRNAs. More intriguing is the observation that many genes are downregulated in common between the 5′ and 3′ isomiRs, but not by the canonical sequence. These two isomiRs have different seed sequences, but share the remaining sequence including the additional two 3′ nt, where they differ from the caninacal miR-140-3p. This suggests defining roles for parts other than the seed sequence in target determination. Perhaps this includes the involvement of the so-called supplemental region (nucleotides ~13–16) of the miRNA that supplements seed interactions [40]. It has also been reported that modifications at the 3′ end are associated with miRNA processing and stability [41]. One could speculate whether this particular modification at the 3′ end of the 3′ isomiR enhanced the isomiR's stability leading to a wider target spectrum compared to the canonical sequence despite the identical seed region.

The usage of primary cells is both a strength and a limitation of this study. Natural donor variability can sometimes be considerable, as seen in Fig. 3, Fig. 5. Cell lines, in contrast, tend to exhibit less data variation. Still, despite the donor variability seen using primary cells, we believe they give a more realistic image of the changes induced by the introduction of miRNAs. This may be particularly important as the 5′ isomiR may be a candidate for therapeutic interventions.

Another limitation is the use of stringent statistical tools in the analysis of big data sets. To avoid false discoveries, stringent compensatory measures are used which may lead to false negative results. The downregulation of a number of genes in the *IFNA/B* cascade and all the HLA class II genes led us to question the apparently unchanged concentrations of *IFNA*, *IFNB* and *CIITA* following miRNA transfection. RT-qPCR analysis showed that *IFNB* was downregulated by all three sequences, *IFNA* was not detected, and *CIITA* was downregulated by all, but most strongly by the 5′ sequence. Also *IFNG* is an example of a gene disqualified from significance due to donor variation. RNA-seq showed that only the 5′ isomiR downregulated *IFNG*, while RT-qPCR confirmed its downregulation by 5′ isomiR as well as by the other two miRNAs. It is therefore important to supplement such big data analysis with more sensitive approches for validation.

## Author contributions

Rua N. Al-Modawi (R.N.A), Tommy A. Karlsen (T.A.K) and Jan E. Brinchmann (J.E.B) conceptualized and designed the study. R.N.A performed all the experiments. All authors analyzed and interpreted the data. R.N.A wrote the original draft and prepared all figures. T.A.K. and J.E.B reviewed and edited the manuscript. J.E.B acquired funding and resources. T.A.K. and J.E.B supervised the project. All authors critically revised the article for important intellectual content and approved the version to be submitted. All authors take responsibility for the integrity of the work as a whole.

## Declaration of competing interest

None of the authors have any conflicts of interest to declare.
